# Learning Experiences in an Intensive Vaccination Training Course in Japan: A Qualitative Study

**DOI:** 10.7759/cureus.78574

**Published:** 2025-02-05

**Authors:** Hirohisa Fujikawa, Mikio Hayashi, Daisuke Son, Masato Eto

**Affiliations:** 1 Center for General Medicine Education, School of Medicine, Keio University, Tokyo, JPN; 2 Department of Medical Education Studies, International Research Center for Medical Education, Graduate School of Medicine, The University of Tokyo, Tokyo, JPN; 3 Center for Health Professions Education, Kansai Medical University, Osaka, JPN; 4 Department of Community-Based Family Medicine, Faculty of Medicine, Tottori University, Yonago, JPN

**Keywords:** clinical procedure, covid-19, pandemic, professionalism, vaccination training course

## Abstract

Introduction: Medical students rarely have opportunities to perform common clinical procedures, and this is especially true in Japan. An intensive vaccination training course was developed to resolve this issue. Medical students experienced (almost) their first experience with needles in a live person with the help of their colleagues and supervisors and seemed to learn various things during the course. However, the details of their learning experiences are not clear; therefore, this study aimed to explore their learning experiences.

Methodology: The research team, comprising a PhD student and experts in health professions education, interviewed 12 course participants to explore their learning experiences. They analyzed anonymized transcripts using inductive thematic analysis within a social constructivist paradigm.

Results: Qualitative analysis showed the following seven themes: (1) changes in clinical clerkships due to the COVID-19 pandemic, (2) recognized entry into the medical professional community, (3) smooth clinical procedure, (4) the vaccination target is a human being, (5) sense of responsibility associated with receiving compensation, (6) working with colleagues, and (7) presence of supervisors. The participants emphasized that working with real vaccinees, rather than mannequins, created pressure not to fail, which positively influenced their learning.

Conclusions: This study revealed that the participants felt a sense of entry into the medical professional community and gained confidence in performing a smooth clinical procedure. Some conditions of the course, such as the vaccination target, working with colleagues, and the presence of supervisors, promoted their learning. The findings will inform international faculty members about the development of curricula for vaccination skills and other clinical procedural skills.

## Introduction

Medical practice involves a wide variety of medical procedures performed on a daily basis. Training in these procedural skills is a critical component of medical education [1]. The Association of American Medical Colleges publication includes proficiency in clinical procedures as one of the 13 Core Entrustable Professional Activities for entry into residency programs [2]. A considerable body of literature has shown that the consensus among clinical educators is that medical undergraduates should be proficient in basic procedural skills upon graduation [1,3,4], and it is now expected that these techniques be mastered in the early stages of medical training.

However, previous studies have reported that medical students have few opportunities to perform common medical procedures during their training, and many lack confidence in performing them [5,6]. In 2013, Dehmer et al. found that medical students performed most of the basic procedural skills infrequently and had a low self-assessment of their ability to do them without assistance [5]. Dickson et al., in their study conducted in 2013, identified a significant discrepancy between the expectations of family medicine residency program directors regarding the procedural skills that medical school graduates would have at the start of their residency and the experience of recent medical school graduates. Notably, postgraduate year 1 residents reported being able to independently perform only 5 of the 15 procedures expected by program directors [6]. These findings indicate that undergraduate trainees in the current medical education system lack both experience and confidence in basic procedural skills. It is, therefore, imperative that universities provide such training in clinical procedures so that trainees can assume responsibility for safe patient care upon entering the profession [1].

In the Japanese undergraduate medical education system, the lack of opportunity for medical students to experience medical procedures during their clinical training has long been an issue [7]. Although a shift from traditional *observational* to *participatory* clinical clerkship was encouraged in the 2000s, the problem of inadequate opportunity has not sufficiently improved. For example, a government survey of medical schools in Japan found that only approximately 20% of medical students performed subcutaneous, intradermal, and intramuscular injections under the guidance and supervision of a supervising physician and that less than 5% were confident enough to perform the procedure independently [7]. In response, the University of Tokyo in Japan initiated an intensive vaccination training course for medical students in 2019, in which fifth- and sixth-year medical students administer flu vaccinations to the staff of the University of Tokyo Hospital. A study conducted at the University of Tokyo showed that medical students gained confidence in their vaccination technique after participating in the course and significantly improved their technique when assessed using a validated assessment tool [8,9]. Interestingly, however, the educational value of the course appeared to extend beyond these immediate benefits. For many of the participants, the course represented a first (or near-first) experience in inserting a needle into a living person - with the help of peers and under the supervision of physicians - which, in turn, appeared to produce a marked post-course invigoration in motivation and enthusiasm for their study and professional future. Accordingly, there was a need to further clarify the educational benefits of this course.

In this qualitative study, we sought to answer the following research questions: (1) What did medical students in the intensive vaccination training course learn from their first (or near-first) experience of inserting needles into a living person? (2) What factors facilitated their learning? We speculated that the findings of this study would be of international interest to faculty members involved in the development of curricula for clinical procedural skills.

## Materials and methods

Study setting

This qualitative study was conducted in Japan in 2021. We followed the Standards for Reporting Qualitative Research recommendations [10]. Recruitment, interviews, and discussion of the data analysis were held online due to the COVID-19 pandemic.

The Japanese standard undergraduate medical education program lasts six years [11]. In the first and second years, students receive preclinical education. They study clinical medicine in the third and fourth years, including internal medicine and surgery. After passing computer-based testing and an objective structured clinical examination, they receive the title *student doctor* and begin clinical clerkships. They take the national licensing examination at the end of the sixth year. Those who pass and aim to practice clinical medicine enter a mandatory two-year postgraduate clinical training program.

The intensive vaccination course of the University of Tokyo was intended for fifth- and sixth-year medical students at the university (i.e., *student doctors*). Details of the intensive vaccination course are given in Table 1. During the course orientation in October 2021, we briefed the participants on the indications, effectiveness, and potential complications of the influenza vaccine. As the influenza vaccine is typically administered subcutaneously in Japan, we focused on this injection route. After the explanation, participants practiced subcutaneous injections using a simulator. In November 2021, we conducted the four-day main portion of the course. The course consisted of one morning session and one afternoon session, each lasting two hours, for a total of eight sessions. Each participant attended one session and then administered the flu vaccine to approximately 20 staff of the University of Tokyo Hospital. Each session consisted of five to seven students. They were divided into groups of two or three, with one member acting as vaccinator and the others as assistants, switching roles as needed to ensure that vaccination opportunities were generally equal. The students helped each other with vaccination techniques and taught each other as needed. The supervising physicians provided guidance as needed. After each session, the medical students and supervising physicians met to reflect on the experience for further learning. Participants were given appropriate financial compensation for their time in providing vaccination services.

**Table 1:** Details of the intensive vaccination course.

Orientation part (October 2021)	Main part (November 2021)
Lecture, Practice subcutaneous injection using a simulator	Vaccinations for hospital staff (Groups of two to three medical students, mutual help among medical students as needed, supervisors provided guidance as needed), Reflection

Research team and reflexivity

Our research team included four people with different academic backgrounds and research experience. The first author was a graduate of the University of Tokyo. At the time of the interviews, he was a PhD student majoring in health professions education at the University of Tokyo. The second author (MD, MHPE, PhD) was a researcher in health professions education and earned his PhD from the University of Tokyo. The third author (MD, MHPE, PhD) was a graduate of the University of Tokyo and an expert in health professions education. The last author (MD, PhD) was a graduate of the University of Tokyo, an expert in health professions education, and the director of the intensive vaccination training course at the university.

The philosophical basis of the study was based on the social constructivist paradigm. Social constructivism is the theory that learning is constituted by the dynamic interaction between the individual and the environment (e.g., other people, objects, and activities that occur there). The theory states that human knowledge is not discovered but socially constructed [12].

Study participants

First, the faculty of the Department of Medical Education Studies invited all fifth- and sixth-year medical students of the University of Tokyo to participate in the vaccine administration training course via email. Given the hospital’s space limitations, we decided to recruit about 50 medical students on a first-come, first-served basis. In the end, 48 medical students applied to participate in the course. Second, all 48 medical students involved in the vaccination course were invited to participate in the study by the first author, a PhD student in the Department of Medical Education Studies; of these, 12 students agreed to participate. A summary of the profiles of these 12 participants is presented in Table 2.

**Table 2:** Characteristics of the study participants.

No.	Sex	Academic level	How many times have you inserted a needle into people as a clinical procedure rather than for practice?
1	Female	Sixth	0
2	Male	Sixth	0
3	Male	Sixth	1
4	Female	Sixth	0
5	Male	Sixth	0
6	Male	Fifth	0
7	Female	Fifth	0
8	Male	Fifth	0
9	Female	Fifth	0
10	Male	Fifth	0
11	Male	Fifth	2-3
12	Male	Fifth	0

Data collection

The first author collected the qualitative data from 12 semi-structured one-on-one interviews via Zoom between December 2021 and June 2022. Interviews lasting 30-60 minutes were performed in Japanese and audiotaped on Zoom. An interview guide was used to examine the research questions (Table 3). In practice, each of the interviews was conducted flexibly, and the study participants could take the discussion in any direction. The audio data were transcribed verbatim.

**Table 3:** Interview guide.

No.	Items
1	In your daily clinical clerkships, how many clinical procedures do you perform? How many times have you inserted needles into people as a clinical procedure rather than for practice?
2	How did you feel about the act of sticking a needle before participating in the course? And has this feeling changed in any way since the first vaccination?
3	What did you learn from this intensive vaccination training course?
4	What factors contributed to the learning?
5	How did you feel about being in the course with your colleagues? Did it affect your learning in any way?
6	How did you feel about having physicians supervise you in the course? Did it affect your learning in any way?

Data analysis

Anonymized transcripts were entered into Microsoft Excel version 16.66.1 and analyzed using thematic analysis, which involved generative coding and theorizing to identify instances in the data set that were conceptually similar [13,14]. Data analysis followed an inductive approach inspired by Braun and Clarke [15]. Inductive thematic analysis is a flexible method for organizing and analyzing patterns in collected data. In this study, we used the following steps: First, the first author read the transcriptions iteratively to familiarize himself with the data. Second, the first author coded the data. Third, the first and second authors repeatedly discussed and reviewed the codes and named the themes. Fourth, all authors discussed the results iteratively and finally reached a consensus. All authors confirmed that they had reached a theoretical saturation, as no additional themes emerged, despite the small sample size [16].

Ethical approval

This study was approved by the Institutional Review Board of the University of Tokyo (2020364NI). All the study participants provided written consent.

## Results

**Figure 1:**
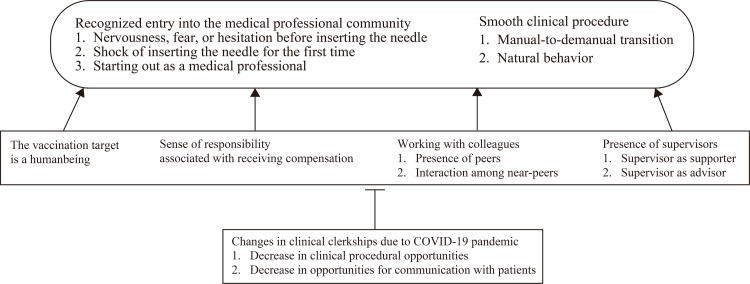
Associations among the emergent themes. Image credit: Hirohisa Fujikawa.

Seven emergent themes were identified from the thematic analysis: (1) changes in clinical clerkships due to the COVID-19 pandemic, (2) recognition of entry into the medical professional community, (3) smooth clinical procedures, (4) the vaccination target being a human, (5) sense of responsibility associated with receiving compensation, (6) collaboration with colleagues, and (7) presence of supervisors. Some themes consisted of several subthemes. Figure 1 shows associations among the emergent themes. Below, we described the details of the themes and subthemes with quotes (numbers identifying each participant corresponded to those in Table 2).

Theme 1: Changes in clinical clerkships due to COVID-19 pandemic

Subtheme 1.1: Decrease in Clinical Procedural Opportunities

Participants reported that the COVID-19 pandemic decreased opportunities to participate in clinical procedures.

“Still, I feel that, compared to the seniors of previous years, I have not had the opportunity to experience something like that (clinical procedure).” (P2, M, 6th)

“Under the influence of the COVID-19 pandemic, I haven’t seen many clinical procedures.” (P5, M, 6th)

Subtheme 1.2: Decrease in Opportunities for Communication With Patients

Some participants mentioned that the COVID-19 pandemic had reduced opportunities to communicate with patients, but that the vaccination course provided them with valuable opportunities.

“Our class was one in which most of the clinical clerkships were done online due to the COVID-19 pandemic, we couldn’t go to the wards much, and our opportunities to interact with patients were quite limited. In that sense, (this vaccination course) was a great and valuable opportunity, especially for our class.” (P1, F, 6th)

Theme 2: Recognized entry into the medical professional community

Subtheme 2.1: Nervousness, Fear, or Hesitation Before Inserting the Needle

In this intensive vaccination training course, most of the participants inserted a needle into a living person (not a mannequin) for the first time. Participants felt nervous or fearful before inserting a needle.

“I had experience sticking needles into mannequins during Objective Structured Clinical Examinations and such, but I had almost never stuck a needle into an actual patient, so I was quite nervous and wondered if I could do it…I was quite nervous and anxious when I actually inserted the needle into the first person.” (P3, M, 6th)

One participant expressed her hesitation regarding whether she was really allowed to perform the clinical procedure, although she was qualified as a *student doctor*.

“I was wondering if I could really do it (the procedure). After all, although I passed the Objective Structured Clinical Examination and Computed Based Testing and became a student doctor, I felt that those examinations were like a game. I wondered if I was really allowed to do the procedure.” (P4, F, 6th)

Subtheme 2.2: Shock of Inserting the Needle for the First Time

Some participants reported that the first time they inserted a needle, they were shocked in a way that would leave a lifelong impression.

“The moment I stuck the needle in the first person was a great shock…I felt like I had finally done it…it was a big milestone for me…It was like the first time I rode a bicycle before entering elementary school, and I think I will always remember the first time I stuck a needle into a person.” (P2, M, 6th)

“It was a dramatic experience to stick a needle for the first time” (P4, F, 6th)

Subtheme 2.3: Starting Out as a Medical Professional

Participants had a sense of increasing medical professionalism as a result of this course.

“It’s a bit childish, but wow, I have finally become a doctor, like I’ve done some medical work. Like, I got to ride a bike for the first time. For example, I would brag to my mother or father that I finally got the flu vaccine myself as part of the program.” (P1, F, 6th)

“Sticking needles into people is not allowed for the general public, but only for medical professionals. I felt it was a responsible job. I think it was a very important thing to do.” (P2, M, 6th)

“I was very nervous at first when I actually inserted the needle. So I thought it was the first step in invasive clinical procedures, or the first step as a medical professional.” (P11, M, 5th)

Several participants expressed that they had a clear vision of their future career path. In particular, Participant 4 expressed confidence in her own future as a physician, noting again that she had few opportunities for clinical practice during the pandemic.

“Of course, I had to do dissections and pass the exams…but of course I felt like a customer during the clinical clerkship…I didn’t really have a clear picture of what it would be like to be a doctor…The first time I stuck a needle was a dramatic experience, so I thought I would become a doctor through a series of experiences like this…I think it was such a big change for me.” (P4, F, 6th)

Participant 9, while referring to herself as “the COVID-19 Generation,” described the confidence he gained from this course.

“I always thought that if we became medical residents with very little clinical experience, we would be treated like ‘the COVID-19 Generation,’ like a generation that cannot do clinical procedures…I think it gives me some confidence to be able to say that I have had that experience.” (P9, F, 5th)

Theme 3: Smooth clinical procedure

Subtheme 3.1: Manual-to-Demanual Transition

Participants reported that from the beginning to the end of the course, they gradually became more aware of things that were not explicitly mentioned in the manual.

“Of course, I got used to actually performing subcutaneous injections, but I also learned how to do the unwritten, invisible procedures, such as immediately discarding the needle and avoiding hesitation…I learned how to do these procedures in my own way, which allowed me to prepare effectively.” (P2, M, 6th)

Subtheme 3.2: Natural Behavior

Several participants described a process by which they became more aware of their natural behavior and practiced approach as healthcare professionals.

“If I was extremely anxious about performing the clinical procedure, I don’t think the patient would feel good either, so I had to act like I could do it, so there was a change in my behavior.” (P8, M, 5th)

“At first…I was really desperate to inject without making mistakes…after injecting about 10 people, it became like a workflow, so I gradually shifted my focus to how smoothly, how leanly, how quickly, and how painlessly I could get the job done.” (P4, F, 6th)

Theme 4: The vaccination target is a human

Participants described the effectiveness of the vaccination target being a person, rather than a simulator, as a factor that enabled the various learning experiences mentioned earlier. One participant highlighted the characteristics of the inoculation target being a human as follows:

“The strength or elasticity of the skin varies greatly from person to person. On the day of the course, I realized that it could be difficult to insert the needle into skin depending on the person.” (P12, M, 5th)

Participants emphasized the importance of performing the clinical procedure on a real person, contrasting it with a simulator.

“During the Objective Structured Clinical Examination, I felt that even if I fail, it was just a mannequin. But for this course, every time was the real person, so I always felt a sense of tension that I could not fail. This tension had a positive effect on me, and I think it helped me learn a lot faster.” (P3, M, 6th)

“When I injected into the arm of a mannequin (instead of a real person), even if I made a mistake, I wouldn’t cause any trouble…In a situation where I really had to succeed, and I did succeed, I could gradually get used to the procedure by accumulating such experiences.” (P10, M, 5th)

Theme 5: Sense of responsibility associated with receiving compensation

In this course, participants were financially compensated for their vaccination work. Some participants expressed their own changes in response to this system.

“Because I was being paid for my work, I should pay the utmost respect and courtesy to the vaccinated people.” (P2, M, 6th)

“I thought that I didn’t need to be paid for my practice, but when I was paid, I felt that I had to do it properly…I probable felt like I had to practice properly, and I think it gave me a little sense of responsibility.” (P4, F, 6th)

Theme 6: Working with colleagues

Subtheme 6.1: Presence of Peers

Several participants talked about the learning that came from comparing themselves with their peers.

“(The peers were) almost all at the same level as me. It was good to work with peers, both in terms of a good example of what not to do and in terms of taking advantage of each other’s good points.” (P4, F, 6th)

Some participants described the stimulation received by seeing their classmates working hard, something they did not see on a daily basis.

“In the (daily) clinical clerkship, we medical students do not perform invasive clinical procedures. There are almost no opportunities for me to see my classmates working really hard and being really nervous.” (P7, F, 5th)

Some participants described the psychological effects of having classmates around.

“We are not used to performing vaccination, so we get nervous…if my classmates had not been there, I would have been even more nervous…I would have been exhausted, so I think it was quite good for me, like a relaxing effect.” (P1, F, 6th)

Subtheme 6.2: Interaction Among Near-Peers

The fifth-year medical students discussed their perspectives on the stimulation they received from the sixth-year students.

“The 6th medical student who was in the booth next to me was particularly chatty and a good talker…I thought that was great and wanted to imitate him…I didn’t have that skill.” (P7, F, 5th)

“The 6th seniors were very reliable…They gave me a lot of advice and were very skillful…I hope to be like them next year.” (P9, F, 5th)

Conversely, sixth-year students also learned from the fifth-year students.

“I could learn that by observing inexperienced clinical procedures…I could see how patients reacted. Seeing that, I might have thought a little bit that I need to fix my skills.” (P2, M, 6th)

Sixth-year students also described the strength their presence represented for the junior fifth-year students as follows:

“I could give advice to the junior medical students, or when they seemed unsure of themselves, I could give them confidence…I learned how to act as a senior student in such a way. As a senior student, I was able to point out details that supervising physicians could not.” (P2, M, 6th)

Theme 7: Presence of supervisors

Subtheme 7.1: Supervisor as a Supporter

Participants spoke of a sense of security due to the presence of a supervisor as a supporter and the learning benefits that this encouraged.

“The effect of the presence of the supervisor was a sense of security. I felt very confident about inoculating…because the doctors were watching me.” (P3, M, 6th)

“I think there’s a considerable amount of anxiety when (medical trainees) are doing these basic clinical procedures for the first time…In a situation where there’s more proper guidance around them…it’s quite a relief.” (P8, M, 5th)

Subtheme 7.2: Supervisor as Advisor

Participants expressed learning motivation that was enhanced by receiving practical advice from supervising physicians.

“I got advice that I should do more of this or that…It was great, and I think I was more motivated, worked harder, and got better when I got specific advice (from supervising physicians).” (P4, F, 6th)

## Discussion

This study found that medical students who underwent an intensive vaccination training course, felt like it was their entry into the medical professional community, and acquired practiced clinical procedure skills. Four factors that made this learning possible were identified: the vaccination target is not a mannequin but a human; a sense of responsibility associated with receiving paid compensation for their vaccination work; working with colleagues (peers and near-peers); and the presence of supervisors. The study also identified the negative impact of the COVID-19 pandemic on the day-to-day clinical clerkships of medical students, which, in turn, further emphasized the educational value of the course.

The theme of *recognized entry into the medical professional community* can be captured in the framework of transition. Transition has recently received substantial attention in the field of medical education. The medical education continuum includes several transitions [17], such as from pre-clinical to clinical education, from undergraduate to postgraduate education, and from residency to specialty training. Transitions may be challenging and a source of anxiety and stress for those who are transitioning [18,19]. Transition can lead to decreased well-being for learners, with increased burnout and suicide risk among learners [20]. Previous studies on the transition from medical undergraduate to resident have found that residents report feeling inadequately prepared for their training and describe a lack of organizational support [21,22]. To address these concerns, internship preparation courses are provided in the senior-year curriculum in many medical schools [23]. Although the course at the University of Tokyo was originally developed for training in vaccination techniques, it was helpful in the broader context of the transition from medical student to resident. In particular, the course seemed to promote professional identity formation (PIF) among medical students through the experience of inserting a needle into a living person for the first time. In 2019, Cruess et al. described the various factors that influence medical trainees’ PIF, one of which was *Symbols and Rituals* [24]. By participating in such activities, medical trainees publicly demonstrate their participation in a community of practice, and this act helps to shape their identity [25]. Typical symbols are white coats, and the white coat ceremony is still widely held throughout the world [26,27]. Wearing a stethoscope and reciting the Hippocratic Oath may also be exemplars [24]. In the present study, the experience of sticking a needle into a person for the first time in the course had special significance, as the quotes in “Starting out as a medical professional” clearly demonstrate. Inserting a needle into a living person might act as a symbol and ritual, prompting the PIF of the medical students.

In addition, whereas previous studies often describe transitions as a struggle [28,29], the experiences of the present course were expressed in positive terms. The structure of this course at the University of Tokyo appeared to allow this, albeit the precise mechanism is unclear. First, the target population for vaccination was humans. Although simulation-based health professional education has progressed, the performance of clinical procedures on real people is considered an irreplaceable experience [5]. The experiential nuances of dealing with living patients are an essential and irreplaceable part of the health professional's education [5]. Indeed, in this study, as Participant 3 indicated, the medical students felt the tension of not being able to fail, which they did not feel during the Objective Structured Clinical Examination because they were dealing with real people rather than mannequins, and this had a positive effect on them. Second, the medical students worked with colleagues in this course. Peer-assisted learning (PAL) is an umbrella term that includes near-peer teaching (i.e., students of a higher year teach those of a lower year) and peer-to-peer teaching. PAL allows medical students to develop their teaching skills as well as to improve their performance [30,31]. While anxiety can easily arise from exposure to new concepts [32], the presence of PALs of peers who are familiar with the student’s issues can reduce the anxiety associated with learning. A previous study indicated that PALs helped students reduce anxiety, become familiar with the ward context, and feel like part of the team [33]. In this study, as some participants indicated, reduced anxiety due to the presence of colleagues leads to effective learning. Third, the presence of supervisors was also important. Medical students usually feel major concerns before performing clinical procedures; these should be allayed by direct observation and detailed feedback by supervisors [34], which may in turn lead to better learning experiences.

We should note that the course and this study were conducted during the COVID-19 pandemic. The narratives of the study participants showed that the pandemic had had various negative consequences on their clinical clerkships, which was consistent with the findings of previous research. During the pandemic, part of medical education was replaced by an online format instead of a face-to-face format, to varying degrees among different countries. While some studies reported that the online format was effective in health professions education [35], several studies indicate its potential negative effects. A Polish study found that online learning was less effective than face-to-face learning in improving clinical skills and social competence, although there was no difference between face-to-face and online learning in improving knowledge [36]. The following disadvantages of online learning in undergraduate medical education have also been reported: lack of hands-on practice [37], lack of communication between learners and educators [38], and visual impairment due to prolonged exposure to screens [39]. In Japan, many universities turned to online lectures during the COVID-19 pandemic. Medical students in their preclinical and even clinical years were required to attend online lectures to avoid contact with high-risk patients [40]. As of writing, however, undergraduate medical education in Japan is conducted in a closely similar way to that before the pandemic. Accordingly, we cannot exclude the possibility that learning experiences in this intensive vaccination training course after the COVID-19 pandemic era differ from those during the pandemic. A comparison of these present results with those of studies exploring the learning experiences of course participants in the current era would provide further insight.

There were some potential limitations of the study. First, it was conducted at a single university in Japan, and the sample size was relatively small; accordingly, the findings of the study may not be transferable. However, as noted in the Introduction, the lack of opportunities for medical undergraduates to perform clinical procedures is an issue that is recognized not only within the University of Tokyo in Japan but also internationally. Determining the transferability of our findings therefore awaits similar courses and studies in other universities in Japan and internationally. Second, the course participants were those who of all medical students expressed a wish to participate; moreover, we interviewed only those course participants who wished to be interviewed. Thus, we cannot exclude the possibility that our participants were originally highly motivated to learn. Our present findings might therefore be expanded if this course is incorporated into the formal university curriculum and the opinions of various medical students are obtained. Third, this study was conducted under a cross-sectional design, and the results may be further expanded by future longitudinal studies.

## Conclusions

The study showed that, although the COVID-19 pandemic significantly decreased the clinical experience of medical students, the participants of this intensive vaccination training course felt like they had entered into the medical professional community and had also acquired practiced clinical procedure experience. Their learning appeared to have been promoted by several conditions of the course, including the vaccination target (a human rather than a mannequin), work with colleagues, and the presence of supervisors. These results may be of interest to faculty members across the world who work in the development of curricula for vaccination skills and other clinical procedural skills.
